# A phase I open-label, dose-escalation study of NUC-3373, a targeted thymidylate synthase inhibitor, in patients with advanced cancer (NuTide:301)

**DOI:** 10.1186/s13046-024-03010-1

**Published:** 2024-04-02

**Authors:** Pavlina Spiliopoulou, Farasat Kazmi, Francesca Aroldi, Thomas Holmes, David Thompson, Lucinda Griffiths, Cathy Qi, Matthew Parkes, Simon Lord, Gareth J. Veal, David J. Harrison, Vicky M. Coyle, Jill Graham, Thomas R. Jeffry Evans, Sarah P. Blagden

**Affiliations:** 1 School of Cancer Sciences, University of Glasgow, Glasgow, UK; 2 Beatson West of Scotland Cancer Centre, Glasgow, UK; 3 Early Phase Clinical Trials Unit, Churchill Hospital, Oxford University Hospitals, Oxford, UK; 4 Department of Oncology, Oncology Clinical Trials Office, University of Oxford, Oxford, UK; 5 Centre for Statistics in Medicine and Oxford Clinical Trials Research Unit (OCTRU), Oxford, UK; 6 Translational and Clinical Research Institute, Newcastle University Centre for Cancer, Newcastle upon Tyne, UK; 7 School of Medicine, University of St Andrews, St Andrews, UK; 8 NuCana plc, 3 Lochside Way, Edinburgh, UK; 9 Patrick G. Johnston Centre for Cancer Research, Queens University Belfast, Belfast, UK; 10 Department of Oncology, University of Oxford, Old Road Campus Research Building, Oxford, OX3 7DQ UK

**Keywords:** NUC-3373, 5-FU, 5-fluorouracil, Fluoropyrimidines, Thymidylate synthase, Resistant cancer

## Abstract

**Purpose:**

5-fluorouracil (5-FU) is inefficiently converted to the active anti-cancer metabolite, fluorodeoxyuridine-monophosphate (FUDR-MP), is associated with dose-limiting toxicities and challenging administration schedules. NUC-3373 is a phosphoramidate nucleotide analog of fluorodeoxyuridine (FUDR) designed to overcome these limitations and replace fluoropyrimidines such as 5-FU.

**Patients and methods:**

NUC-3373 was administered as monotherapy to patients with advanced solid tumors refractory to standard therapy via intravenous infusion either on Days 1, 8, 15 and 22 (Part 1) or on Days 1 and 15 (Part 2) of 28-day cycles until disease progression or unacceptable toxicity. Primary objectives were maximum tolerated dose (MTD) and recommended Phase II dose (RP2D) and schedule of NUC-3373. Secondary objectives included pharmacokinetics (PK), and anti-tumor activity.

**Results:**

Fifty-nine patients received weekly NUC-3373 in 9 cohorts in Part 1 (*n* = 43) and 3 alternate-weekly dosing cohorts in Part 2 (*n* = 16). They had received a median of 3 prior lines of treatment (range: 0–11) and 74% were exposed to prior fluoropyrimidines. Four experienced dose-limiting toxicities: two Grade (G) 3 transaminitis; one G2 headache; and one G3 transient hypotension. Commonest treatment-related G3 adverse event of raised transaminases occurred in < 10% of patients. NUC-3373 showed a favorable PK profile, with dose-proportionality and a prolonged half-life compared to 5-FU. A best overall response of stable disease was observed, with prolonged progression-free survival.

**Conclusion:**

NUC-3373 was well-tolerated in a heavily pre-treated solid tumor patient population, including those who had relapsed on prior 5-FU. The MTD and RP2D was defined as 2500 mg/m^2^ NUC-3373 weekly. NUC-3373 is currently in combination treatment studies.

**Trial registration:**

Clinicaltrials.gov registry number NCT02723240. Trial registered on 8th December 2015. https://clinicaltrials.gov/study/NCT02723240.

## Introduction

Fluoropyrimidines, such as 5-fluorouracil (5-FU), are widely utilized anti-cancer agents that can be given as monotherapy or more commonly in combination therapy for the treatment of a range of tumor types, including colorectal, stomach, head and neck, pancreatic, and breast cancers [1]. The anti-cancer activity of 5-FU, and its other forms floxuridine (FUDR) and capecitabine (Xeloda®), is largely attributed to the active metabolite, fluorodeoxyuridine-monophosphate (FUDR-MP or FdUMP), which inhibits the enzyme thymidylate synthase (TS), a critical enzyme in de novo nucleotide synthesis and cell survival [2]. However, due to multiple limitations, 5-FU is not efficiently converted to FUDR-MP. More than 85% of administered 5-FU is degraded by the enzyme dihydropyrimidine dehydrogenase (DPD) in the liver; therefore, most of the drug is catabolized before it has an opportunity to exert any therapeutic effect [3]. 5-FU also requires the presence of nucleobase transporters for intracellular transfer and, once inside the cell, requires complex enzymatic processing to generate FUDR-MP [4]. These key limitations impacting breakdown, transport, and activation have been linked to poorer outcomes in patients receiving 5-FU [5]. Additionally, the generation of RNA-directed metabolites by fluoropyrimidines, such as fluorouridine triphosphate (FUTP), is responsible for a number of dose-limiting toxicities (DLTs), including neutropenia, diarrhoea and mucositis, impacting patient safety [6].

Although various strategies have been investigated to counter these limitations, such as biochemical modulation with leucovorin (LV) to enhance TS binding or increased infusion durations, none have successfully addressed these shortcomings in a comprehensive manner. NUC-3373, a phosphoramidate nucleotide analog, is the first such agent specifically designed to overcome the limitations and pharmacologic challenges associated with 5-FU and other fluoropyrimidines [7]. NUC-3373 is a pre-activated and protected form of FUDR, one of a new class of anti-cancer agents (ProTides) being developed to improve efficacy, safety and pharmacokinetic profiles of nucleoside analog drugs. ProTides contain a phosphate that is protected by a phosphoramidate group (consisting of an aryl, an ester, and an amino acid) with unique structural properties that stabilize the compound and protect it against enzymatic breakdown.

**Fig. 1:**
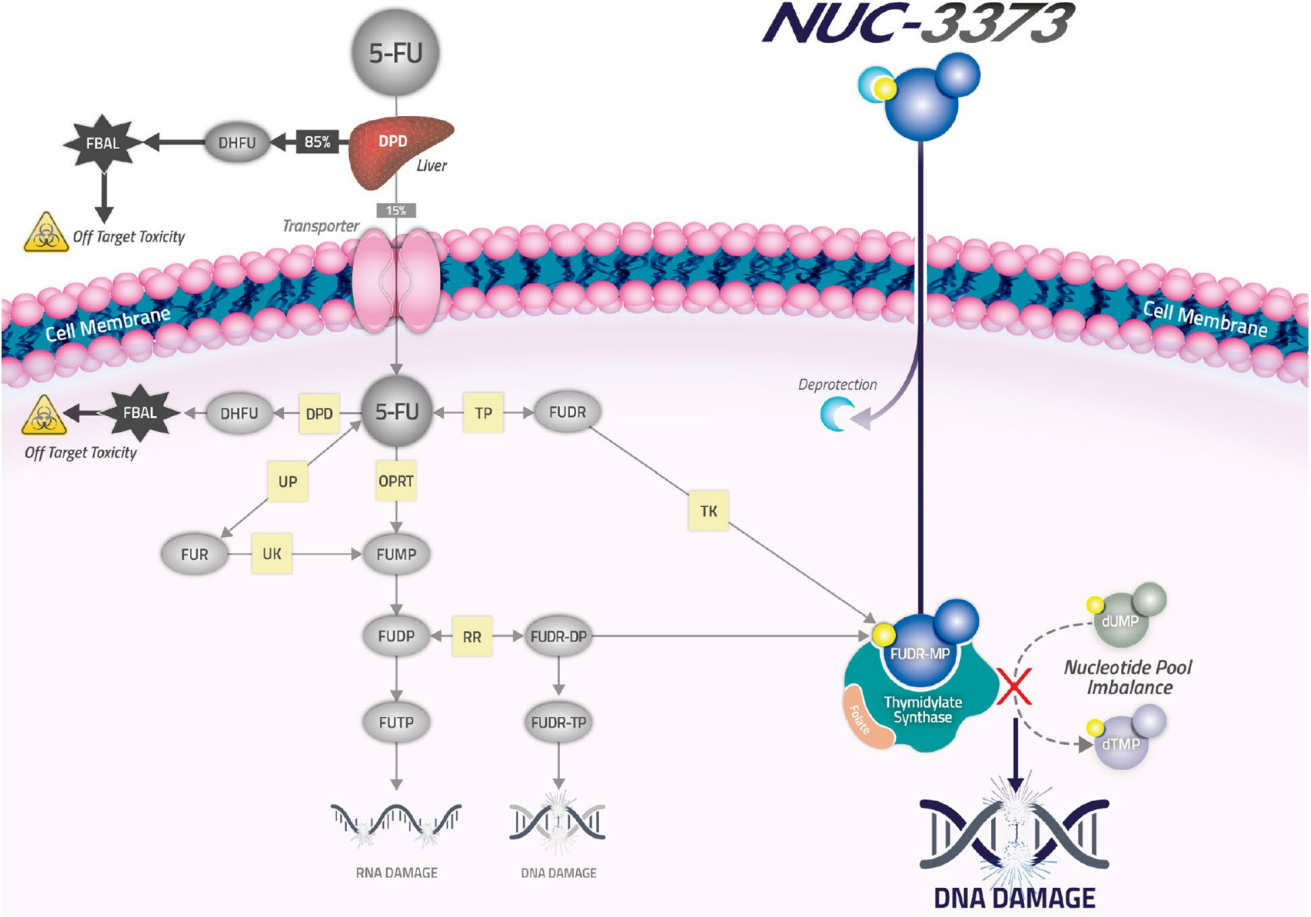
NUC-3373 and 5-fluorouracil mechanisms of action. NUC-3373 is a pre-activated and protected form of FUDR. It carries a phosphoramidate group which protects the FUDR-MP moiety, making it resistant to breakdown by DPD and allowing it to enter cancer cells without the need for nucleobase transporters. As NUC-3373 contains the active metabolite, FUDR-MP, it does not require phosphorylation by TK. These properties would confer NUC-3373 with more stability than 5-FU, resulting in a longer half-life and the generation of higher intracellular levels of FUDR-MP, making it a more potent inhibitor of TS than 5-FU. Abbreviations: 5-FU = 5-fluorouracil; DPD = dihydropyrimidine dehydrogenase; DHFU = dihydrofluorouracil; dTMP: deoxythymidine monophosphate; dUMP: fluorodeoxyuridine-monophosphate; FBAL = alpha-fluoro-beta-alanine; FUDR = fluorodeoxyuridine; FUDR-MP = fluorodeoxyuridine monophosphate; FUDR-DP = fluorodeoxyuridine diphosphate; FUDR-TP = fluorodeoxyuridine triphosphate; FUMP = fluorouridine monophosphate; FUDP = fluorouridine diphosphate; FUTP = fluorouridine triphosphate; FUR = fluorouridine; OPRT = orotate phosphoribosyl transferase; RR = ribonucleotide reductase; TK = thymidine kinase; TP = thymidine phosphorylase; UK = uridine kinase; UP = uridine phosphorylase

Preclinical studies have shown that NUC-3373 is resistant to breakdown by DPD [8] and does not require nucleobase transporters to enter cancer cells [9]. As NUC-3373 contains the active anti-cancer metabolite FUDR-MP, it does not rely on phosphorylation by thymidine kinase (TK) [8, 9]. As a result of these properties, NUC-3373 generates substantially higher intracellular levels of FUDR-MP and is a more potent inhibitor of TS than 5-FU in vitro, while generating markedly lower levels of the toxic metabolite FUTP (Fig. 1) [10]. In human colorectal cancer mouse xenograft models, NUC-3373 showed greater tumor inhibition than 5-FU [8].

Here we describe the first-in-human study of NUC-3373 given as monotherapy to patients with advanced solid tumors (NuTide:301). The study was conducted to determine the MTD, schedule, and recommended Phase II dose (RP2D) of NUC-3373 and to assess its safety, pharmacokinetics (PK) and anti-tumor activity.

## Patients and methods

### Study design and objectives

This Phase I, open-label, two-part, dose-escalation study was designed to evaluate the safety, PK, and anti-cancer activity of single-agent NUC-3373, in addition to establishing its MTD, schedule and RP2D, in patients with advanced solid tumors. The primary objective was to determine the MTD and RP2D when given weekly on Days 1, 8, 15 and 22 (Part 1) or alternate weekly on Days 1 and 15 (Part 2) of 28-day cycles, until disease progression or unacceptable toxicity. In standard treatment, 5-FU has typically been administered on a weekly or alternate weekly schedule. Therefore, both schedules were selected for this study in order to allow for an efficient characterization of each schedule’s ability to produce sustained drug exposures that may be associated with biologic effect, while addressing safety questions. Secondary objectives included safety and tolerability, PK, and evaluation of anti-tumor activity through assessment of disease control and tumor response. Patients were enrolled sequentially into dose-escalation cohorts using a modified 3 + 3 design. The study was conducted at three clinical centres in the United Kingdom: the Churchill Hospital, Oxford; The Beatson West of Scotland Cancer Centre, Glasgow; and the Patrick G. Johnston Centre for Cancer Research, Belfast. The study was performed in accordance with the Declaration of Helsinki and the principles of Good Clinical Practice [11]. The protocol was approved by the South Central - Oxford C Research Ethics Committee [15/SC/0644] and all patients provided written informed consent before undergoing any study procedures.

### Patients

Patients ≥18 years of age with a histologically-confirmed solid tumor not amenable or refractory to standard therapies, or for which standard therapy did not exist, were eligible for the study. Other inclusion criteria included an Eastern Cooperative Oncology Group (ECOG) performance score of 0–2, adequate organ function, measurable disease on radiological imaging (in accordance with Response Evaluation Criteria in Solid Tumors [RECIST] v1.1), and/or evaluable disease [12]. Exclusion criteria included prior allergy or cardiac events attributed to 5-FU or capecitabine, active hepatitis B, C or HIV and symptomatic central nervous system metastases.

### Treatment

NUC-3373 was administered via central or peripheral venous access device as an infusion lasting 30 minutes (for doses ≤750 mg/m^2^), 2 hours (for doses ≤1500 mg/m^2^) or 4 hours (for doses > 1500 mg/m^2^). A starting dose of 125 mg/m^2^ NUC-3373 was selected for the first 3 patients (Part 1) and was escalated sequentially in cohorts of 3 to 6 patients using a modified 3 + 3 design. Dose-escalation stopped when the MTD was determined. The MTD was defined as the highest dose level for which fewer than 2 out of 6 (or < 33%) patients experienced DLTs. Following recruitment to the third dose group in Part 2, the Trial Management Group (TMG) agreed that this schedule would be stopped, and recruitment would instead resume in Part 1, to determine the RP2D for the weekly schedule. This decision was partially based on the Part 1 safety profile, which indicated that weekly dosing was tolerable. Thus, the weekly schedule was investigated as a priority to maximize dose intensity and potential activity. Furthermore, as the NUC-3373 starting dose and escalations at each step were conservative, the number of dose-escalation cohorts required in Part 1 was higher than anticipated and the planned sample size was reached before commencing Part 2. This, along with delay to recruitment during the COVID-19 pandemic, prolonged the duration of the study which was closed prior to opening of any dose expansion cohorts at the RP2D. Dose intensity (DI) was calculated for each patient per cycle using actual DI/allocated DI, where allocated DI was [4 x dose assigned (mg/m^2^)] / 28 days for the weekly schedule and [2 x dose assigned (mg/m^2^)] / 28 days for the alternate weekly schedule, and the actual DI was [sum of all doses received in a cycle (mg/m^2^)] / [Duration of cycle in days including any delays before starting].

### Assessment

All patients who received at least one dose of NUC-3373 were included in the safety analysis population. Safety parameters were continually assessed and based on treatment-emergent adverse events (TEAEs), graded according to the National Cancer Institute Common Terminology Criteria for Adverse Events version 4.03 [13], along with clinical laboratory data and physical examinations. Due to the association of 5-FU to cardiac toxicity, echocardiograms and electrocardiograms (ECG) were conducted at baseline, at the end of study participation and during the study if clinically indicated. Patients were defined as evaluable for DLT assessment if they had received at least 75% (Part 1) or 100% (Part 2) of the intended treatment with NUC-3373 during Cycle 1 or if they experienced a DLT. A DLT was defined as any of the following occurring during the first treatment cycle: Grade 3 or higher non-haematological toxicity (excluding nausea/vomiting/diarrhoea or rash that responded to standard medical treatment), Grade 3 or higher infusion reaction, Grade 3 or higher nausea/vomiting/diarrhoea or rash that occurred despite standard medical treatment, Grade 4 neutropenia, febrile neutropenia (any grade), Grade 4 thrombocytopenia, any toxicity related to NUC-3373 that was unresolved after a treatment delay of > 14 days, or isolated/recurrent toxicity that was judged by the Investigators to be a DLT.

Tumor response assessments were performed for the safety population. Computed tomography (CT) based tumor assessments were conducted according to RECIST 1.1 [12] at screening and every 8 weeks until progression. Responses from all available post-baseline scans were used for derivation of the best overall response (BOR) and disease control rate (DCR). A response of stable disease was only considered if the scan was taken at least 6 weeks after the baseline scan. The highest reduction of the sum of diameters of target lesions was selected and reported for each patient with available post-baseline scan. This was expressed as a percentage change from baseline. Median progression free survival was estimated in the safety population using the Kaplan-Meier method, event times were from date of cycle 1 day 1, a PFS event was all progressions or death if death occurred without progression. The restricted mean survival time (RMST) for progression-free survival is also reported.

Plasma NUC-3373 concentrations were measured using validated liquid chromatography tandem mass spectrometry (LC-MS/MS) methods and standard PK parameters were calculated using a non-compartmental analysis approach with Certara Phoenix 8.3 WinNonlin software. PK parameters were generated from available data for a subset of study patients. Blood samples were collected at set timepoints on Cycle 1 Day 1 and Cycle 1 Day 15 with timepoints dependent on the NUC-3373 infusion duration. For those who received NUC-3373 over 30 minutes to 2 hours (doses < 1500 mg/m^2^), PK samples were collected pre-dose, at end of infusion, and at 15, 30 and 45 mins, and 1, 1.5, 2, 4, 6, 24 and 48 h after the end of infusion. For those who received NUC-3373 over 4 hours (doses ≥1500 mg/m^2^), samples were collected pre-dose and at 45 mins and 2, 4, 4.5, 4.75, 5, 6, 7, 8, 24 and 48 h after the start of infusion.

## Statistical analysis

Descriptive analyses were carried out using STATA 16.1 (StataCorp. 2019. Stata Statistical Software: Release 16. College Station, TX: StataCorp LLC). Median (range) and numbers (with percentages) were used to summarize continuous and categorical variables, respectively. Sample size calculations were based on a Fleming design [14]. No formal statistical analyses were planned or performed on safety, PK or efficacy data. With respect to primary objectives and endpoints, no specific hypotheses were tested statistically. The primary focus was on determining the MTD and RP2D, evaluating the safety and PK profiles, and identifying a range of biologically active doses in patients with advanced cancers.

## Data availability

The data generated in this study are available upon request to researchers who provide a methodologically sound proposal to the corresponding author. To gain access, data requestors will need to sign a data access agreement.

## Results

### Patient characteristics

**Fig. 2:**
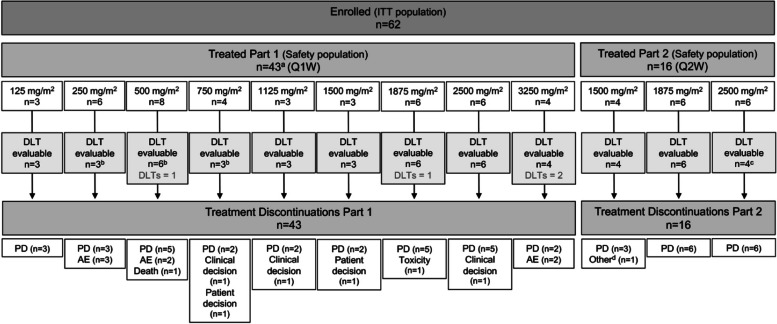
Patient disposition. ^a^A total of 46 patients were enrolled in Part 1; however, 3 patients (1 patient each in the 125, 250 and 2500 mg/m^*2*^ cohorts) did not receive any study treatment with NUC-3373. ^b^Six patients in Part 1 were not evaluable for DLT assessment: 5 patients withdrew in Cycle 1 (3 patients in the 250 mg/m^*2*^ cohort and 2 patients in the 500 mg/m^*2*^ cohort) and 1 patient in the 750 mg/m^*2*^ cohort received less than 3 doses of NUC-3373 (the required 75% of Cycle 1 doses). ^c^Two patients in Part 2 were not evaluable for DLT assessment: 1 patient withdrew in Cycle 1 and 1 patient received less than 2 doses of NUC-3373, both in the 2500 mg/m^*2*^ cohort.^d^ One patient discontinued at the discretion of the Investigator due to declining performance status (ECOG 3). Abbreviations: AE = adverse event; DLT = dose-limiting toxicity; ITT = intention-to-treat; PD = progressive disease; Q1W = weekly; Q2W = alternate weekly

Between 22 February 2016 and 13 October 2020, 100 patients were screened for the study and 62 eligible patients were enrolled. Of the 62 patients in the intention-to-treat (ITT) population, 59 received at least one dose of NUC-3373 and were included in the safety analysis population. A total of 43 patients were treated across 9 dosing cohorts in Part 1 (weekly) and 16 patients were treated across 3 dosing cohorts in Part 2 (alternate weekly), as shown in Fig. 2. Overall, 51 patients were evaluable for DLT assessment, including 37 patients in Part 1 and 14 patients in Part 2. At the time of database lock (06 August 2021), no patients remained on study, all having discontinued treatment. The most common reasons for treatment discontinuation were progressive disease (75%) and adverse events (12%).

Patient baseline characteristics in the ITT population are presented in Table 1. Across both parts of the study, patients had a median age of 59.5 years (range 20–77) and all had a good performance status (48% ECOG PS 0 and 52% PS 1). The most common tumor types were cancers of the colon (31%), rectum (15%), oesophageal/gastroesophageal junction (10%), pancreas (7%) and stomach (5%), and the majority of patients had a histology of adenocarcinoma (74%). Patients had received a median of 3 prior lines of treatment (range 0–11). The majority of patients (74%) had received prior therapy with fluoropyrimidines, with 52% of patients having received 5-FU, 52% capecitabine, and 15% tipiracil/trifluridine.

**Table 1:** Patient baseline characteristics

Characteristic	Part 1 (*n* = 46)	Part 2 (*n* = 16)	Total (*n* = 62)
Median age, years [range]	59.5 [20, 77]	59.5 [20, 77]	59.5 [20, 77]
Sex, *n* (%)			
Female	18 (39)	7 (44)	25 (40)
Male	28 (61)	9 (56)	37 (60)
Histological type, *n* (%)			
Adenocarcinoma	35 (76)	11 (69)	46 (74.2)
Adenosquamous Carcinoma	0	1 (6)	1 (1.6)
Alveolar Rhabdomyosarcoma	0	1 (6)	1 (1.6)
Basal Cell Carcinoma	0	1 (6)	1 (1.6)
Low Grade Endometrioid Adenocarcinoma	0	1 (6)	1 (1.6)
Mesothelioma	1 (2)	0	1 (1.6)
Metastatic Melanoma	1 (2)	0	1 (1.6)
Sarcoma	1 (2)	0	1 (1.6)
Spindle Cell Carcinoma	1 (2)	0	1 (1.6)
Squamous Cell	4 (9)	0	4 (6.5)
Undifferentiated	0	1 (6)	1 (1.6)
Histology not specified	3 (7)	0	3 (4.8)
Performance Status, *n* (%)			
0	23 (50)	7 (44)	30 (48)
1	23 (50)	9 (56)	32 (52)
Cancer Type, *n* (%)			
Colon	16 (35)	3 (19)	19 (31)
Rectum	8 (17)	1 (6)	9 (15)
Oesophageal/gastroesophageal junction	3 (7)	3 (19)	6 (10)
Pancreas	2 (4)	2 (13)	4 (7)
Cervix	1 (2)	1 (6)	2 (3)
Other	16 (35)	6 (38)	22 (35)
Prior therapies			
Prior systemic therapy, median [range]	3 [0, 6]	3 [1, 11]	3 [0, 11]
Prior fluoropyrimidine therapy ^a^, *n* (%)	37 (80)	9 (56)	46 (74)
Prior 5-FU	27 (59)	5 (31)	32 (52)
Prior capecitabine	26 (57)	6 (38)	32 (52)
Prior tipiracil / trifluridine	7 (15)	2 (13)	9 (15)
Median BSA, m^2^ [range]	1.84 [1.4, 2.6]	1.88 [1.5, 2.4]	1.86 [1.4, 2.6]

*BSA* body surface area

^a^Prior fluoropyrimidine therapy includes systemic 5-FU and/or capecitabine and/or tipiracil/trifluridine

### Treatment exposure

Patient exposure to NUC-3373 in Parts 1 and 2 is presented in Table 2. Across all cohorts, patients received a median of 2 cycles of treatment, with a median duration of exposure of 8 weeks, in both parts of the study. A higher proportion of patients had dose modifications (70% vs 50%) or dose reductions (19% vs 6%) in Part 1 than in Part 2.

**Table 2:** NUC-3373 treatment exposure

**Characteristic**	**Part 1 Cohorts (Q1W)**								
	**125 mg/m**^**2**^ **(*n*** **= 4)**	**250 mg/m**^**2**^ **(*n*** **= 7)**	**500 mg/m**^**2**^ **(*n*** **= 8)**	**750 mg/m**^**2**^ **(*n*** **= 4)**	**1125 mg/m**^**2**^ **(*n*** **= 3)**	**1500 mg/m**^**2**^ **(n = 3)**	**1875 mg/m**^**2**^ **(*n*** **= 6)**	**2500 mg/m**^**2**^ **(*n*** **= 7)**	**3250 mg/m**^**2**^ **(*n*** **= 4)**
Started treatment, *n*	3	6	8	4	3	3	6	6	4
Did not start treatment, *n*	1	1	0	0	0	0	0	1	0
Median duration of exposure, weeks [range]	7.3 [7.3, 8.1]	4.8 [2, 7.7]	8 [2, 16]	7.5 [6.9, 11]	18.9 [17, 48]	15 [8.1, 41]	8.7 [4.1, 16.3]	8.1 [4.9, 16]	9.9 [2.3, 17.9]
Median completed cycles, *n* [range]	2 [1.8, 2]	1.1 [0.3, 2]	1.9 [0.5, 4]	1.9 [1.8, 2.5]	4 [3.3, 11.8]	3.5 [2, 9.3]	2.1 [1, 4]	2 [1.3, 4]	2 [0.5, 4.3]
Patients completing 6 cycles, *n* (%)	0	0	0	0	1 (33.3)	1 (33.3)	0	0	0
Patients with a dose modification ^a^, *n* (%)	1 (33.3)	5 (83.3)	5 (62.5)	3 (75)	2 (66.7)	2 (66.7)	3 (50)	5 (83.3)	4 (100)
Patients with a dose reduction, *n* (%)	0	0	1 (12.5)	2 (50)	1 (33.3)	0	1 (16.7)	0	3 (75)
**Characteristic**	**Part 2 Cohorts (Q2W)**								
	**1500 mg/m**^**2**^ **(*n*** **= 4)**			**1875 mg/m**^**2**^ **(*n*** **= 6)**			**2500 mg/m**^**2**^ **(*n*** **= 6)**		
Started treatment, *n*	4			6			6		
Did not start treatment, *n*	0			0			0		
Median duration of exposure, weeks [range]	12.2 [6.9, 44.6]			7.9 [6.3, 16.9]			6.9 [3, 8.9]		
Median completed cycles, *n* [range]	3 [1.5, 9.5]			2 [1.5, 4]			1.75 [0.5, 2]		
Patients completing 6 cycles, *n* (%)	1 (25)			0			0		
Patients with a dose modification ^a^, *n* (%)	3 (75)			2 (33.3)			3 (50)		
Patients with a dose reduction, *n* (%)	1 (25)			0			0		

*Q1W* weekly, *Q2W* alternate weekly

Percentages are based on the number of patients in the safety population (started treatment)

^a^Dose modifications include missed, reduced or delayed doses

### Safety

Patients were sequentially recruited into the study as shown in Fig. 2. Of the 59 patients in the safety population, 51 (86%) were evaluable for DLT assessment, including 37/43 patients (86%) in Part 1 and 14/16 patients (88%) in Part 2. In the Part 1 weekly dosing cohorts, 4 patients (9%) had DLTs: one patient each in the 500 mg/m^2^ and 1875 mg/m^2^ cohorts had DLTs of raised Grade 3 transaminases; 1 patient in the 3250 mg/m^2^ cohort had a DLT of Grade 2 headache; and 1 patient in the 3250 mg/m^2^ cohort had a DLT of Grade 3 hypotension (transient). Based on this, the MTD and RP2D was defined as 2500 mg/m^2^ NUC-3373 given weekly. The MTD was not reached in Part 2 as recruitment to this schedule was stopped after the third dose group due to sample size restrictions and prioritization of the weekly schedule to maximize dose intensity and potential activity. No DLTs were observed in Part 2.

In Part 1, all 43 patients in the safety analysis population experienced TEAEs. The most common Grade 1–2 TEAEs observed across all dose cohorts were fatigue (58%), diarrhoea (42%), nausea (42%), infusion-related reaction (35%), constipation (33%), and abdominal pain (26%). The most common Grade 1–2 TEAEs that were considered related to NUC-3373 were fatigue (54%), nausea (37%), infusion-related reactions (35%), and diarrhoea (33%) (Table 3). The most common Grade 3 TEAEs were fatigue (9%), intravascular device-related infection (5%), ALT increased (5%), blood alkaline phosphatase increased (5%), and transaminases increased (5%). The most common Grade 3 TEAEs that were considered related to NUC-3373 were increased ALT (5%) and transaminases (5%). No Grade 4 TEAEs were reported.

**Table 3:** Treatment-related adverse events

SOC PT	Part 1 (*n* = 43)		Part 2 (*n* = 16)	
	Grade 1–2 *n* (%)	Grade 3–4 *n* (%)	Grade 1–2 *n* (%)	Grade 3–4 *n* (%)
**Blood and lymphatic system disorders**				
Anaemia	6 (13.9)	0	3 (18.8)	0
Platelet count decreased	3 (6.9)	0	0	0
**Cardiac disorders**				
Palpitations	0	0	1 (6.3)	0
Tachycardia	0	0	1 (6.3)	0
**Gastrointestinal disorders**				
Nausea	16 (37.2)	0	6 (37.5)	0
Diarrhoea	14 (32.6)	0	5 (31.3)	0
Vomiting	6 (13.9)	0	4 (25.0)	0
Constipation	4 (9.3)	0	3 (18.8)	0
Abdominal pain	2 (4.7)	0	1 (6.3)	0
Dyspepsia	0	0	1 (6.3)	0
Flatulence	0	0	1 (6.3)	0
**General disorders and administration site conditions**				
Fatigue	23 (53.5)	1 (2.3)	7 (43.8)	0
Pyrexia	4 (9.3)	0	0	0
Mucositis	3 (6.9)	0	0	0
Chest pain	0	1 (2.3)	1 (6.3)	0
Gait disturbance	0	0	1 (6.3)	0
**Infections**				
Herpes zoster	0	0	0	1 (6.3)
**Injury, poisoning and procedural complications**				
Infusion-related reaction	15 (34.9)	0	6 (37.5)	0
**Investigations**				
ALT increased	4 (9.3)	2 (4.7)	2 (12.5)	0
AST increased	4 (9.3)	1 (2.3)	1 (6.3)	1 (6.3)
ALT and AST increased	4 (9.3)	2 (4.7)	0	0
Blood ALP increased	2 (4.7)	0	0	0
Blood bilirubin increased	2 (4.7)	0	0	0
GGT increased	0	1 (2.3)	0	0
Hepatic enzyme increased	0	0	0	1 (6.3)
**Metabolism and nutrition disorders**				
Hyperglycaemia	3 (6.9)	0	0	0
Decreased appetite	2 (4.7)	0	4 (25.0)	0
Hypophosphatemia	2 (4.7)	0	0	0
**Musculoskeletal and connective tissue disorders**				
Myalgia	2 (4.7)	0	0	0
Muscle spasms	0	0	1 (6.3)	0
**Nervous system disorders**				
Dysgeusia	7 (16.3)	0	0	0
Headache	4 (9.3)	0	2 (12.5)	0
Neuropathy peripheral	4 (9.3)	0	0	0
Lethargy	3 (6.9)	0	1 (6.3)	0
Posterior reversible encephalopathy syndrome	0	1 (2.3)	0	0
**Respiratory, thoracic and mediastinal disorders**				
Dyspnoea	3 (6.9)	0	1 (6.3)	0
Throat irritation	2 (4.7)	0	0	0
Dyspnoea exertional	0	0	1 (6.3)	0
Dysphonia	0	0	1 (6.3)	0
**Skin and subcutaneous tissue disorders**				
Palmar-plantar erythrodysesthesia syndrome	2 (4.7)	0	0	0
Rash	0	0	1 (6.3)	0
**Vascular disorders**				
Flushing	5 (11.6)	0	0	0
Hypotension	0	1 (2.3)	0	0

*ALP* alkaline phosphatase, *ALT* alanine aminotransferase, *AST* aspartate aminotransferase, *n* number of patients, *PT* preferred term, *SOC* system organ class

Percentages are based on the total number of patients in the respective safety population

A total of 15 patients (35%) had serious adverse events (SAEs), 9 (21%) of whom had Grade 3 SAEs and one (2%) of whom had a Grade 5 AE (unrelated to NUC-3373) of pulmonary haemorrhage. Five patients (12%) had treatment-related SAEs, 2 patients with chest pain attributed to coronary vasospasm, 1 with pyrexia, 1 with drug hypersensitivity, and 1 with posterior reversible encephalopathy syndrome (PRES).

In Part 2, all 16 patients in the safety analysis population experienced TEAEs. The most common Grade 1–2 TEAEs observed across all dose cohorts were fatigue (56%), diarrhoea (44%), nausea (44%), vomiting (38%), anaemia (38%), and infusion-related reactions (38%). The most common Grade 1–2 TEAEs that were considered related to NUC-3373 were fatigue (44%), nausea (38%), infusion-related reactions (38%), and diarrhoea (31%) (Table 3). Recurring infusion related reactions were mitigated with prophylactic treatment with antihistamine and steroid treatment prior to subsequent dosing. The most common Grade 3–4 TEAE reported was anaemia (13%). All other Grade 3–4 TEAEs were reported in only 1 patient (6%) each. Two patients had Grade 3 TEAEs that were considered related to NUC-3373, 1 patient with herpes zoster and 1 patient with increased hepatic enzymes and aspartate aminotransferase (AST) increased. No Grade 4 TEAEs were considered to be related to NUC-3373. Of note, one patient had an infusion reaction to study drug preventing further treatment. The reaction was graded as G2 but the treatment was discontinued as per patient’s preference.

A total of 7 patients (44%) had SAEs, 5 (31%) of whom had Grade 3 SAEs and one (6%) of whom had a Grade 4 SAE of hypercalcemia. Two patients had treatment-related SAEs, one with herpes zoster infection and another with increased hepatic enzymes.

#### Pharmacokinetics

Pharmacokinetic parameters were calculated from individual plasma NUC-3373 concentrations following IV administration of 500–3250 mg/m^2^ NUC-3373 on a weekly or alternate weekly schedule. Thirty-one patients had evaluable PK samples: 500 mg/m^2^ weekly (*n* = 5), 1125 mg/m^2^ weekly (*n* = 3), 1875 mg/m^2^ weekly (*n* = 6), 1875 mg/m^2^ alternate weekly (*n* = 3), 2500 mg/m^2^ weekly (*n* = 6), 2500 mg/m^2^ alternate weekly (*n* = 5), and 3250 mg/m^2^ weekly (*n* = 3). A dose-proportional increase in NUC-3373 Area Under the Curve (AUC) was observed across the concentration range assessed (Table 4).

**Table 4:** NUC-3373 plasma PK parameters on Day 1 and Day 15

Dose / infusion duration/ Regimen	Parameter	C_max_ (mg/mL)	AUC_last_ (h*mg/L)	AUC_(0-∞)_ (h*mg/L)	T_1/2_ (h)	AUC_last_D_ (h*mg/L/mg)	AUC_(0-∞)_D_ (h*mg/L/mg)
**Day 1**							
**500 mg/m**^**2**^ **30 mins Q1W**	N	5	5	5	5	5	5
	Geometric mean	32.54	56.68	70.83	12.21	0.057	0.072
	Geo lower 1SD	22.47	33.08	44.05	4.65	0.033	0.044
	Geo upper 1SD	47.14	97.12	113.89	32.06	0.100	0.117
**1125 mg/m**^**2**^ **2 hours Q1W**	N	3	3	3	3	3	3
	Geometric mean	38.66	72.31	77.47	12.51	0.038	0.041
	Geo lower 1SD	29.65	47.40	51.31	9.61	0.026	0.028
	Geo upper 1SD	50.39	110.31	116.97	16.27	0.056	0.061
**1875 mg/m**^**2**^ **4 hours Q1W**	N	6	6	6	6	6	6
	Geometric mean	25.50	105.59	111.25	8.74	0.032	0.033
	Geo lower 1SD	18.36	60.27	60.67	3.43	0.019	0.019
	Geo upper 1SD	35.43	184.97	204.01	22.28	0.052	0.058
**1875 mg/m**^**2**^ **4 hours Q2W**	N	3	3	3	3	3	3
	Geometric mean	29.05	123.84	125.49	3.39	0.038	0.038
	Geo lower 1SD	26.01	96.77	97.22	1.13	0.033	0.033
	Geo upper 1SD	32.44	158.47	161.98	10.19	0.044	0.045
**2500 mg/m**^**2**^ **4 hours Q1W**	N	6	6	6	6	6	6
	Geometric mean	38.11	172.47	174.21	3.77	0.038	0.038
	Geo lower 1SD	28.80	116.50	116.72	1.74	0.024	0.024
	Geo upper 1SD	50.44	255.33	260.01	8.17	0.059	0.060
**2500 mg/m**^**2**^ **4 hours Q2W**	N	5	5	5	5	4	4
	Geometric mean	43.48	201.76	204.93	5.91	0.046	0.047
	Geo lower 1SD	30.64	124.32	124.14	4.46	0.027	0.026
	Geo upper 1SD	61.71	327.44	338.27	7.85	0.081	0.083
**3250 mg/m**^**2**^ **4 hours Q1W**	N	3	3	3	3	3	3
	Geometric mean	35.50	150.98	151.75	3.71	0.024	0.024
	Geo lower 1SD	22.46	90.69	91.38	3.05	0.014	0.014
	Geo upper 1SD	56.11	251.36	251.99	4.53	0.041	0.041
**Day 15**							
**1875 mg/m**^**2**^ **4 hours Q1W**	N	6	6	6	6	6	6
	Geometric mean	29.68	126.38	130.22	5.59	0.040	0.041
	Geo lower 1SD	21.13	88.19	86.37	3.64	0.025	0.025
	Geo upper 1SD	41.68	181.11	196.34	8.59	0.063	0.068
**1875 mg/m**^**2**^ **4 hours Q2W**	N	3	3	3	3	3	3
	Geometric mean	27.05	116.39	117.87	2.82	0.036	0.036
	Geo lower 1SD	22.09	73.36	73.74	0.97	0.024	0.025
	Geo upper 1SD	33.13	184.66	188.41	8.22	0.052	0.053
**2500 mg/m**^**2**^ **4 hours Q1W**	N	6	6	6	6	6	6
	Geometric mean	39.65	170.48	176.93	4.47	0.037	0.039
	Geo lower 1SD	28.58	112.15	113.84	1.93	0.023	0.024
	Geo upper 1SD	55.02	259.17	275.01	10.32	0.060	0.063
**2500 mg/m**^**2**^ **4 hours Q2W**	N	3	3	3	3	3	3
	Geometric mean	37.36	177.94	180.17	4.95	0.040	0.040
	Geo lower 1SD	23.39	91.64	91.69	4.54	0.021	0.021
	Geo upper 1SD	59.64	345.51	354.00	5.40	0.076	0.078
**3250 mg/m**^**2**^ **4 hours Q2W**	N	2	2	1	1	2	1
	Geometric mean	36.92	136.67	164.37	4.59	0.021	0.026
	Geo lower 1SD	32.91	105.64	–	–	0.016	–
	Geo upper 1SD	41.42	176.83	–	–	0.028	–

*AUC*_*(0-∞)*_ area under the plasma concentration-time curve from time 0 to infinity, *AUC*_*(0-∞)_D*_ dose normalized AUC_(0-∞)_, *AUC*_*last*_ area under the plasma concentration-time curve from time 0 to time of the last quantifiable concentration, *AUC*_*last_D*_ dose normalized AUC_last_, *C*_*max*_ maximum plasma concentration, *Q1W* weekly, *Q2W* alternate weekly, *T*_*1/2*_ half-life.

Geo lower and Geo upper 1 Standard deviation (SD) – 68% range of the geometric mean

The last observed concentrations were between 24 and 48 hours for most patients. The estimated half-life of NUC-3373 showed some inherent variability and ranged from 3 to 10 hours with a geometric mean of 5.61 hours, which is considerably longer than the reported half-life of 5-FU (8–14 minutes). Limited samples were available at the later timepoints, which impacted the typical terminal elimination half-life; however, a longer half-life was observed in patients who had a later PK sample collection timepoint. When comparing dose normalized exposure, all treatment regimens were comparable regardless of whether they were weekly or alternate weekly, with a difference of less than 15% in all comparisons based on the geometric mean ratios. Comparisons of the Day 1 and Day 15 geometric mean ratios were also similar regardless of whether they were weekly or alternate weekly and did not exceed a 15% difference.

#### Efficacy

Radiological response to treatment was determined according to RECIST v1.1 using CT scans conducted every 8 weeks and compared to baseline scans. Of the 43 patients in the Part 1 safety population, 16 had a BOR of stable disease resulting in a DCR of 37% and 18 (42%) had progressive disease (Table 5). When considering only the 34 patients who had at least one post-baseline scan, a DCR of 47% was observed. Of the 16 patients in the Part 2 safety population, 4 had a BOR of stable disease resulting in a DCR of 25% and 10 (63%) had progressive disease (Table 5). When considering only the 14 patients who had at least one post-baseline scan, a DCR of 29% was observed. Across both parts of the study, the overall DCR was 34% in the safety population and 42% in the population who had at least one post-baseline scan. No complete or partial responses were observed in this population of patients who had exhausted all other treatment options.

**Table 5:** Anti-tumor activity of NUC-3373

	**Part 1 Cohorts**									
	**125 mg/m**^**2**^ **(*n *= 3)**	**250 mg/m**^**2**^ **(*n*** **= 6)**	**500 mg/m**^**2**^ **(*n*** **= 8)**	**750 mg/m**^**2**^ **(*n*** **= 4)**	**1125 mg/m**^**2**^ **(*n*** **= 3)**	**1500 mg/m**^**2**^ **(*n*** **= 3)**	**1875 mg/m**^**2**^ **(*n*** **= 6)**	**2500 mg/m**^**2**^ **(*n*** **= 6)**	**3250 mg/m**^**2**^ **(*n*** **= 4)**	**Total (*n*** **= 43)**
**Best overall response, n (%)**										
Complete response	0	0	0	0	0	0	0	0	0	0
Partial response	0	0	0	0	0	0	0	0	0	0
Stable disease	0	0	3	1	3	2	2	2	3	16 (37.2)
Progressive disease	3	3	2	2	0	1	4	3	0	18 (41.9)
**Response rate**^**a**^	**0**	**0**	**0**	**0**	**0**	**0**	**0**	**0**	**0**	**0**
**Disease control rate**^**b**^	**0**	**0**	**3 **	**1 **	**3 **	**2 **	**2 **	**2 **	**3 **	**16 (37.2)**
	**Part 2 Cohorts**									
	**1500 mg/m**^**2**^ **(*n*** **= 4)**			**1875 mg/m**^**2**^ **(*n*** **= 6)**		**2500 mg/m**^**2**^ **(*n*** **= 6)**			**Total (*n*** **= 16)**	
**Best response, n (%)**										
Complete response	0			0		0			0	
Partial response	0			0		0			0	
Stable disease	3			1		0			4 (25)	
Progressive disease	1			5		4			10 (62.5)	
**Response rate**^**a**^	**0**			**0**		**0**			**0**	
**Disease control rate**^**b**^	**3 **			**1 **		**0**			**4 (25)**	

*CR* complete response, *PR* partial response, *SD* stable disease

^a^ Response rate confirmed CR + confirmed PR

^b^ Disease control rate confirmed CR + confirmed PR + confirmed SD

**Fig. 3:**
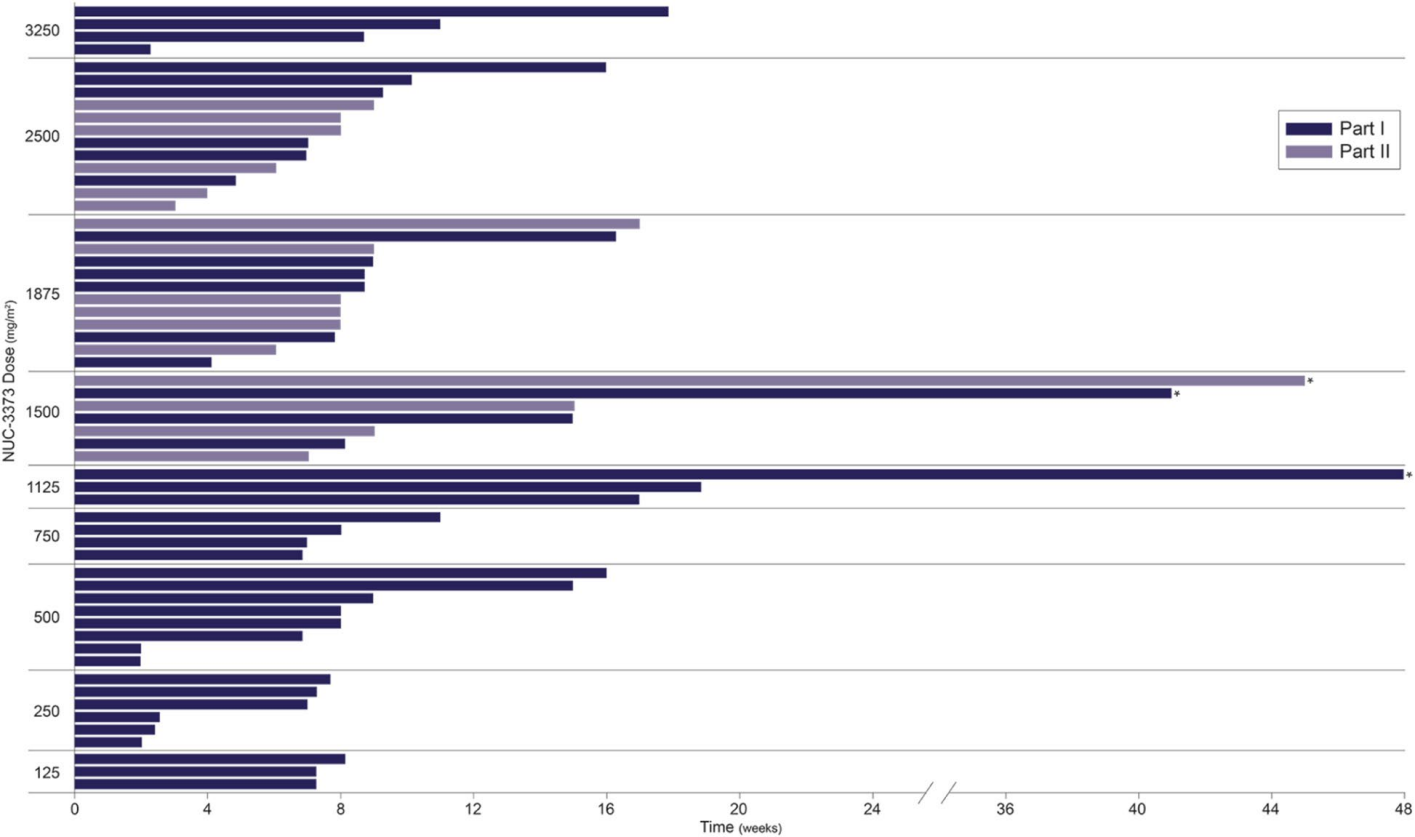
Patient time on treatment. *Details presented in text. Part 1 = weekly NUC-3373 dosing on Days 1, 8, 15 and 22 of 28-day cycles. Part 2 = alternate weekly NUC-3373 dosing on Days 1 and 15 of 28-day cycles

The overall median duration of exposure was 8 weeks across both parts of the study. In Part 1, 10 patients remained on treatment for ≥3 months, with 2 of these patients completing ≥9 months of treatment (Fig. 3). One of the patients completing ≥9 months had colorectal cancer and had received 6 prior lines of therapy, including 5 prior lines of fluoropyrimidine-based therapy (progressed within 2 months on third-line treatment with capecitabine plus oxaliplatin, progressed within 8 months on fourth-line treatment with FOLFIRI, and progressed within 3 months on fifth-line treatment with tipiracil/trifluridine). This patient received 1500 mg/m^2^ NUC-3373 weekly and achieved stable disease for 9 months before choosing to suspend treatment to go on an extended vacation. The other patient completing ≥9 months had cholangiocarcinoma and progressed within 6 months of first-line therapy (gemcitabine in combination with cisplatin). This patient received 1125 mg/m^2^ NUC-3373 weekly and achieved stable disease for 11 months.

**Fig. 4:**
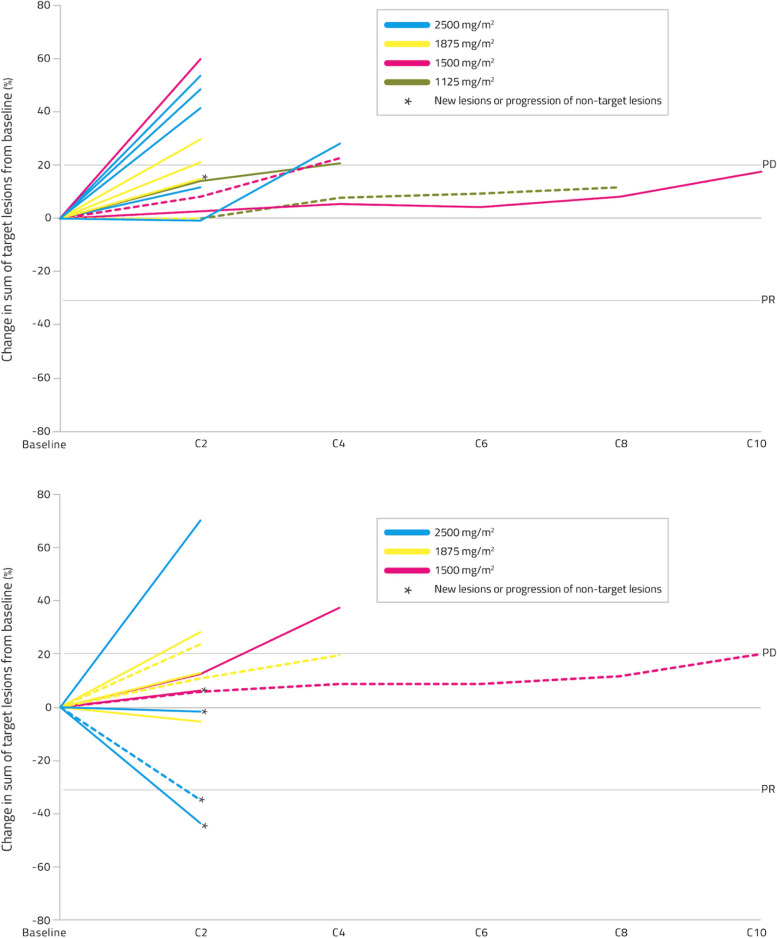
Spider plots depicting tumor responses to treatment. Solid lines represent patients with prior fluoropyrimidine exposure. Dashed lines represent fluoropyrimidine-naïve patients. Upper panel: In Part 1, 18 patients received doses of NUC-3373 between 1125 and 2500 mg/m^2^. Fourteen of these patients had post-baseline scans and are represented above. Lower panel: In Part 2, 16 patients received doses of NUC-3373 between 1500 and 2500 mg/m^2^. Twelve of these patients had post-baseline scans and are represented above

In Part 2, three patients remained on treatment for ≥3 months, with 1 of these patients completing ≥9 months (Fig. 3). This patient had metastatic basal cell carcinoma, had received 2 prior lines of therapy, and had progressed within 3 months of the last line. This patient received 1500 mg/m2 NUC-3373 every 2 weeks and achieved stable disease for 10 months. For part 1, estimated median progression free survival was 7.1 weeks (95% CI 7.0 to 14.7 weeks). The restricted mean progression-free survival time was 12.9 weeks (95% CI 8.8 to 16.9 weeks). For part 2, estimated median progression free survival was 6.9 weeks (95% CI 6.0 to 7.1 weeks). The restricted mean progression-free survival time was 10.8 weeks (95% CI 5.3 to 16.4 weeks). Visualisation of patients’ responses over time is shown in Fig. 4. It is worth noting that the majority of the patients in this study received lower doses than the established RP2D and this Phase I study was not designed to examine efficacy per se.

## Discussion

NUC-3373 is a phosphoramidate modification of FUDR specifically designed to overcome the limitations and pharmacologic challenges associated with 5-FU and other fluoropyrimidines. In this first-in-human clinical study, single-agent NUC-3373 demonstrated a favorable safety and PK profile, compared to historical 5-FU data, in patients with advanced solid tumors. NUC-3373 also showed encouraging signs of anti-tumor activity in heavily pre-treated patients, including those who had progressed on multiple lines of prior fluoropyrimidine-based combination therapies.

In this study, NUC-3373 was well-tolerated at doses up to the MTD of 2500 mg/m^2^ weekly. The most common treatment-related TEAEs were fatigue, nausea, infusion-related reactions and diarrhoea; most of which are also commonly observed with 5-FU. These were mitigated with supportive medications; prophylactic administration of steroid and antihistamine treatment was introduced prior to subsequent dosing for patients with infusion related reactions. Interestingly, other typical 5-FU-related adverse events (such as neutropenia, mucositis and hand-foot syndrome) were either not observed in those receiving NUC-3373 or were observed at a lower incidence and reduced severity when compared with historical data for 5-FU or capecitabine [15, 16]. These findings are consistent with nonclinical data indicating that NUC-3373 generates lower levels of toxic by-products [10], which are implicated in hand-foot syndrome as well as 5-FU-induced hematological and gastrointestinal toxicities [6].

Across both parts of the study, a total of 7 patients (12%) experienced treatment-related SAEs. The SAEs observed were consistent with the overall TEAE profile and with the type of adverse reactions typically associated with 5-FU, including the rare SAE of PRES which was experienced by one patient in this study and was considered to be possibly related to NUC-3373.

The calculated half-life of NUC-3373 in this study was 3–10 hours, shorter than previously reported [17] which is likely due to limited sampling at later timepoints impacting the determination of its terminal elimination half-life. Nonetheless, the longer half-life of NUC-3373 compared to 5-FU (8–14 minutes) would prolong the exposure of tumor cells to FUDR-MP, potentially resulting in enhanced activity of NUC-3373 and enabling shorter administration schedules (1–4 hours, depending on the selected dose) compared to the prolonged infusion times (up to 48 hours) required for 5-FU. A nested PK/PD analyses was conducted on 16/17 patients recruited in the first 3 dose levels [18] demonstrating that within 1 hour of infusion, FUDR-MP, the active metabolite of NUC-3373, was present within thymidylate synthase ternary complexes leading to depletion of the intracellular deoxythymidine monophosphate (dTMP) pool after 2–4 hours. Overall, NUC-3373 PK showed no tendency of non-linearity and the dose-related PK parameters increased with increasing doses. The PK profile of NUC-3373 was comparable on different days of administration and was similar regardless of whether the administration schedule was weekly or alternate-weekly.

The assumed lack of dependency on DPD for the metabolism of NUC-3373 may favour its use in the estimated 5% population who have *DPYD* gene mutations and are at heightened risk of toxicity from standard fluoropyrimidines. However, DPD testing was not conducted on participants in the NuTide:301 study, and it was not routinely conducted at the time of the study. As 74% study patients had received, and tolerated, multiple prior fluoropyrimidine-containing regimens, it is unlikely that any were DPD-deficient.

Patients who received NUC-3373 at the MTD (2500 mg/m2) or below were able to maintain treatment intensity across both the weekly (Part 1) and alternate weekly (Part 2) dosing schedules, with patients in Part 1 generally achieving higher dosing intensities for longer durations. This was associated with a higher median DCR in Part 1 than in Part 2 for both the safety analysis population (37% vs 25%) and a larger number of patients remaining on study beyond one post-baseline scan (47% vs 29%). Notably, the highest DCRs were observed in the 1125 mg/m^2^ (100%), 1500 mg/m^2^ (67%), and 3250 mg/m^2^ (75%) weekly cohorts and the 1500 mg/m^2^ alternate weekly cohort (75%), in which patients remained on treatment for a median of 19, 15, 10, and 12 weeks, respectively, compared to the overall median of 8 weeks. Although there were no RECIST partial or complete responses observed, a number of patients across both parts of the study had prolonged stable disease (SD). This was encouraging given that NUC-3373 was administered as monotherapy and that participants were heavily pre-treated, 74% having previously received 5-FU containing chemotherapy regimens.

The main limitations of this study were the small sample size and the heterogenous nature of the patient population, who had a wide variety of tumor types and had received a range of prior treatment regimens. The NUC-3373 starting dose was conservatively low which resulted in a higher number of dose-escalation steps than initially anticipated; therefore, the planned total recruitment was reached prior to embarking on a Part 2 dose expansion at the RP2D. This was conducted as a single-arm study, and while comparisons with historical 5-FU data may be informative, future studies should include a direct randomized comparison of NUC-3373 versus 5-FU.

In conclusion, NUC-3373 has demonstrated favorable safety, tolerability, pharmacokinetics and clinical activity in patients with advanced solid tumors who have exhausted standard treatment options. These findings position NUC-3373 as a more tolerable alternative to conventional 5-FU that can be administered more conveniently. NUC-3373 is currently being evaluated in combination with other agents in ongoing clinical studies [NCT05678257 and NCT05714553].

## Data Availability

The study was designed by the lead investigators and the sponsor (University of Oxford). The sponsor collected and analysed the data in conjunction with the authors. The datasets during and/or analysed during the current study available from the corresponding author on reasonable request.
